# 3D Bioprinted cancer models: Revolutionizing personalized cancer therapy

**DOI:** 10.1016/j.tranon.2021.101015

**Published:** 2021-01-22

**Authors:** Robin Augustine, Sumama Nuthana Kalva, Rashid Ahmad, Alap Ali Zahid, Shajia Hasan, Ajisha Nayeem, Lana McClements, Anwarul Hasan

**Affiliations:** a Department of Mechanical and Industrial Engineering, College of Engineering, Qatar University, 2713 Doha, Qatar; b Biomedical Research Center (BRC), Qatar University, PO Box 2713 Doha, Qatar; c Department of Biotechnology, St. Mary's College, Thrissur, 680020, Kerala, India; d School of Life Sciences, Faculty of Science, University of Technology Sydney, 2007, NSW, Australia

**Keywords:** Cancer, Cancer models, 3D cancer models, Bioprinting, Personalized medicine

## Abstract

After cardiovascular disease, cancer is the leading cause of death worldwide with devastating health and economic consequences, particularly in developing countries. Inter-patient variations in anti-cancer drug responses further limit the success of therapeutic interventions. Therefore, personalized medicines approach is key for this patient group involving molecular and genetic screening and appropriate stratification of patients to treatment regimen that they will respond to. However, the knowledge related to adequate risk stratification methods identifying patients who will respond to specific anti-cancer agents is still lacking in many cancer types. Recent advancements in three-dimensional (3D) bioprinting technology, have been extensively used to generate representative bioengineered tumor *in vitro* models, which recapitulate the human tumor tissues and microenvironment for high-throughput drug screening. Bioprinting process involves the precise deposition of multiple layers of different cell types in combination with biomaterials capable of generating 3D bioengineered tissues based on a computer-aided design. Bioprinted cancer models containing patient-derived cancer and stromal cells together with genetic material, extracellular matrix proteins and growth factors, represent a promising approach for personalized cancer therapy screening. Both natural and synthetic biopolymers have been utilized to support the proliferation of cells and biological material within the personalized tumor models/implants. These models can provide a physiologically pertinent cell–cell and cell–matrix interactions by mimicking the 3D heterogeneity of real tumors. Here, we reviewed the potential applications of 3D bioprinted tumor constructs as personalized *in vitro* models in anticancer drug screening and in the establishment of precision treatment regimens.

## Highlights

• This review describes how 3D bioprinting can be used for developing patient specific cancer models.
• Bioprinted cancer models containing patient-derived cancer and stromal cells is promising for personalized cancer therapy screening
• 3D bioprinted constructs form physiologically relevant cell–cell and cell–matrix interactions.
• Bioprinted cancer models mimic the 3D heterogeneity of real tumors.

## Graphical abstract

**Figure:**
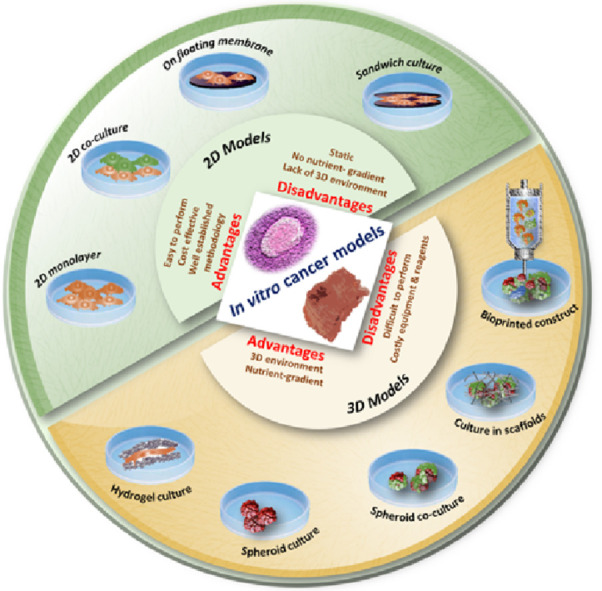

## 1 Introduction

Despite extensive research, the survival and the quality of life with certain types of cancer are still poor, accounting for millions of deaths worldwide [1], [2], [3]. The response to cancer treatments varies substantially between patients and it is associated with significant economic burden, particularly in developing countries [4,5]. Considerable progress has already been made in developing novel therapeutic interventions for cancer including immunotherapies, therapeutic peptides [6], Notch targeted strategies [7] and other targeted therapies that have transformed the field of oncology. The gold standard treatment for the vast majority of cancers includes chemotherapeutic agents; however, the response to these treatments is still variable amongst patients, particularly for poorly characterized cancers. Hence, the “one‐size‐fits‐all” treatment approach can have limited effectiveness, identifying a clinical need for more personalized/precision treatment regimens specific to an individual or a subset of patients. In addition to the overall response, drug efficacy can also vary considerably even across different regions of the tumor [8,9]. This is likely due to tumor heterogeneity, the presence of cancer stem cells and cancer cell plasticity [10,11], indicating the need for a combination of multiple drug treatments including targeted therapies. In support of this, a study investigating various treatment options for 15 different tumor types demonstrated that patients who were treated with targeted therapy had a significantly better overall response rate and progression free survival [12]. Similarly, determining the most suitable therapeutic dose and timing of the treatment, is key in managing cancer patients without debilitating adverse effects.

Unlike i*n vivo* models*, in vitro* cancer models are simplified approaches to study cancer mechanisms and behavior, and to examine the effects of established and novel anti-cancer agents. It is now well established that the soluble factors released from cancer and stromal cells can influence the cell viability/proliferation, cell-cell adhesion, cell migration, mechanotransduction, and signaling of cells within the tumor tissue, which is difficult to replicate in traditional 2D cell culture models. Recent developments have demonstrated that tumors can successfully grow across the 3D microenvironment/extracellular matrix (ECM), resulting in gradient exposure of cancer cells to oxygen and nutrients [13]. Hypoxia or low oxygen can lead to excessive cell proliferation within tumor tissue; highly proliferative cancer cells can generate local hypoxia within tumors under *in vivo* conditions increasing the percentage of non-proliferating viable hypoxic tumor and/or cancer stem cells [14]. These features of tumor tissue are not recapitulated in 2D monolayer cultures [15] and hence 3D cancer models have better physiological relevance for testing drug treatments and understanding disease mechanisms. Amongst the recent 3D models, utilization of spherical models has shown the most promise, which in combination with appropriate microenvironment and biomaterials could revolutionize *in vitro* personalized drug screening. The most frequently utilized 3D cancer models for drug testing include multicellular tumor spheroid model (MCTS), multilayered cell cultures, organotypic slices of cancer tissue, and cell seeded scaffolds [16]. Over the past few decades, printing technology has progressed from 2D printing to an additive process capable of producing 3D shapes. Recently, 3D bioprinting, an additive manufacturing spinoff technology, has been successfully used in laboratories worldwide to create pulsating 3D tissue constructs [17]. The bioprinting field has had substantial technological advances in the last five years becoming the most promising approach for developing 3D constructs of tumor tissue that can be used as models for studying cancer biology and screening anticancer agents [18]. The major advantage of bioprinting is the ability to precisely control and define the desired structure of the tissue construct according to the 3D design [19]. Unlike other approaches for developing 3D cancer models, multiple cells (both cancer and normal) can be deposited with microscale precision by 3D bioprinting, therefore closely reconstituting a cancer microenvironment [18]. Many researchers have been successful in developing bioprinted breast [20], brain [21], skin [22], pancreatic [23], and other cancer models for this purposes.

In this review, we provide a comprehensive summary of the collective findings in relation to various bioprinted cancer models utilized for chemotherapeutic drug screening. 2D and 3D cancer models are critically evaluated and comprehensively compared, in terms of their ability to recapitulate physiological tumors and their microenvironment. Various strategies used for bioprinting of 3D cancer models including inkjet, micro extrusion, and laser ablation technologies as well as cancer and stromal bioinks, and biomaterials, are discussed. This review clearly outlines current challenges and prospects for 3D bioprinting technologies in cancer research by focusing on the clinical application of these technologies for chemotherapeutic drug screening and the development of personalized treatment regimens for cancer patients.

## 2 Precision anticancer drug screening

Cancer patients display a high degree of inter-patient variation in terms of clinical outcomes, prognosis, and response or tolerance to medication [24]. Thus, the need for prognostic preclinical models capable of identifying the most suitable treatment regimens for individual patients is growing rapidly. This personalized approach could enable better response to treatment with reduced incidence of adverse effects [25,26]. The response to chemotherapy treatments depends on molecular subtypes of tumors, cancer stage, comorbidities, genomic background and patient's tolerance to treatments, which can vary significantly between patients. One of the effective approaches to develop personalized treatment is to replicate the disease in laboratory using 3D cell models based on patient-derived tumors following debulking surgery, and test various treatment options for the specific cancer phenotype. While this approach can test the effectiveness of the treatment, it might not be able to determine the adverse and off target, effects. Precision medicine approach does not rely on “one-fits-all” model but rather investigates specific therapeutic interventions suitable for each individual patient that the conventional and general *in vivo* and *in vitro* models cannot be necessarily used for. Patient-derived cell-based tumor organoids and xenografts have shown some promise in advancing precision medicine, enabling the development of personalized chemotherapeutic regimens [27]. Developments in this field resulted in the utilization of bioprinting for anticancer drug screening by generating physiologically relevant 3D cancer or tumor models hence providing a platform of controlled environment and physiologically relevant models [28].

Therefore, testing of multiple chemotherapeutic drugs on patient-derived bioprinted cancer models could identify the most effective combination of chemotherapeutic drug candidates for a individual patient. These bioprinted cancer models can also complement human clinical trials where the effectiveness and mechanisms of drug candidates can be tested on individual basis taking into the account not just the molecular subtype of the tumor but also the differences in age, sex and ethnicity as well as identifying the most optimal doses of chemotherapeutic agents [29].

## 3 Two- and three-dimensional cell culture systems in cancer research

2D cancer models have contributed significantly to our basic understanding of cancer biology over the past several decades. These simple cancer models have also played a significant role in pharmaceutical testing. However, considerable differences in the molecular signaling, protein and gene expression, cell phenotype, cell migration, cell viability, cell proliferation and drug response, have been observed between 2D and 3D cancer models [5,30,31]. 3D systems, especially those based on cell-seeded hydrogel platforms, secrete cytokine and angiogenic factors for longer period of time than a monolayer 2D culture [32,33]. Also, the cell secreted biomolecules tend to be displaced during media replacement hence limiting the ability of a 2D cell culture system to provide the most optimal environment for cells to grow [34]. Furthermore, the lack of adequate ECM to support and communicate with cancer cells can also impact on biological relevance of these *in vitro* systems [35]. Although 2D cell models can offer some insight into cancer biology, the applicability of the results should be interpreted with caution as molecular and cellular interactions are not recapitulated, limiting our understanding of the cell–cell and cell–ECM interactions.

**Fig. 1:**
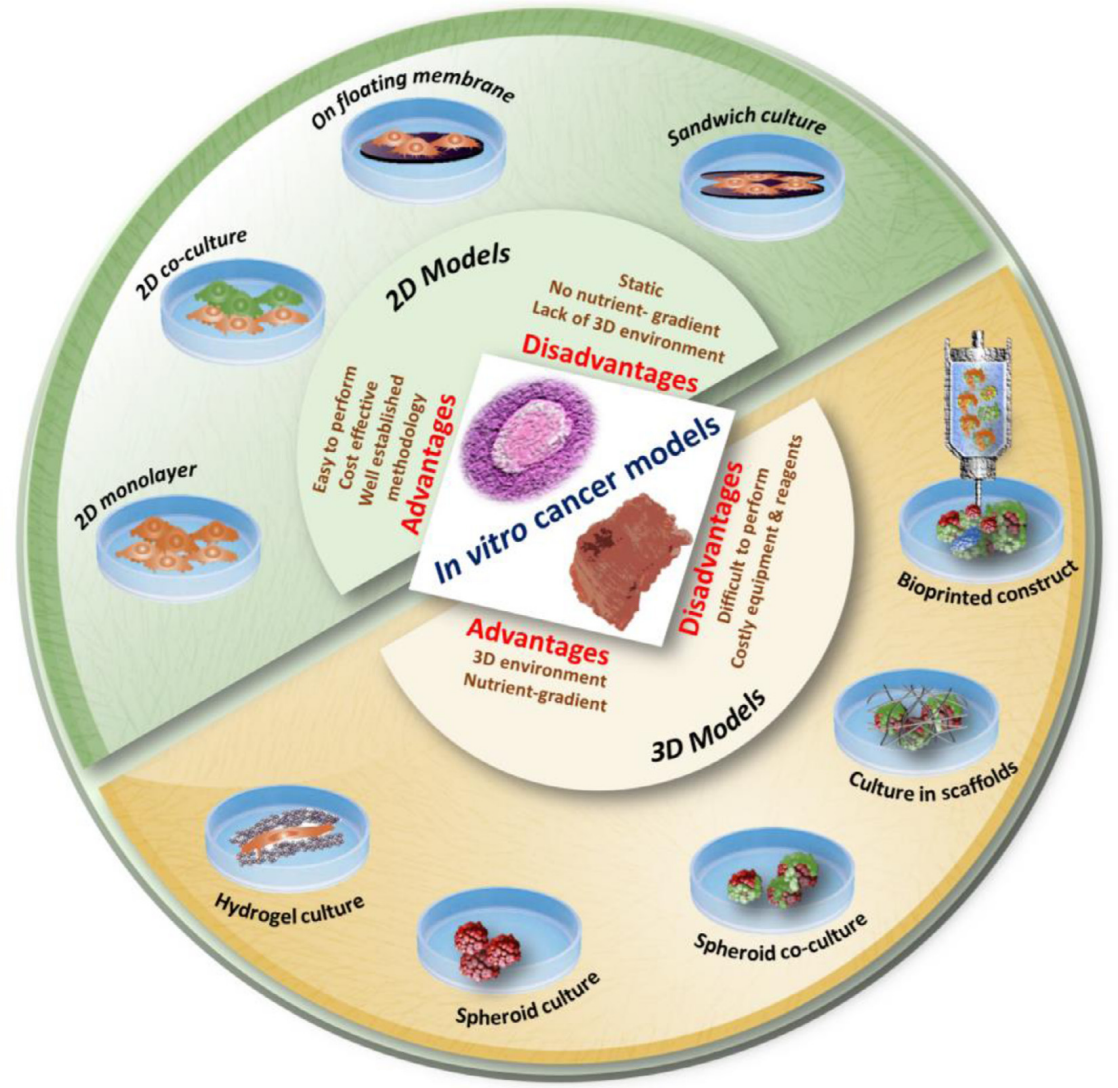
Various *in vitro* cancer models used in chemotherapeutic screening. Evolution of cell-culture models from simple 2D to complex 3D bio-printed models. Conventional 2D monolayer culture, monolayer co-culture, cells grown over floating membranes, and cell monolayer sandwiched between membranes, are the commonly used 2D cancer models in research and drug screening. Cancer cells cultured in hydrogels, spheroid monoculture, spheroid co-culture, cancer/stromal cells cultured in porous 3D scaffolds, and advanced bioprinted constructs are amongst the available 3D cancer models.

On the other hand, 3D cancer constructs represent physiological cancer tissues better, in terms of tumor microenvironment and cellular behavior with characteristic spatial distribution of cells [36]. This has, therefore, contributed significantly to the development of more reliable cancer models that can be used for the screening of potential anticancer agents and novel treatment regimens for various types of cancers [20]. Histopathological analyses of the tumors have confirmed that tumors are 3D structures composed of heterogeneous population of cells including cancer cells, cancer stem cells and healthy cells embedded within stromal tissue [37]. Despite the large body of evidence, which supports the knowledge that 3D models are more physiologically relevant than 2D cultures [38,39], current pharmaceutical screening heavily relies on cells cultured as monolayers due to low cost and ease of use. A number of research studies have assessed the performance of human cells in 2D or 3D culture systems to *in vivo* models. For example, 3D cell refinements within organoids have shown potential as an alternative method to study drug pharmacokinetics within the tumor tissue therefore addressing the gap in transition between 2D cell culture and animal models [40]. These cell models can be overall divided into: (i) co-culture systems, (ii) multicellular spheroids, and (iii) cells loaded or cultured in an ECM mimetic biopolymeric scaffold [41,42] (Fig. 1). Within 3D cell models, both primary cancer cells isolated from patient tumor biopsies or established cell lines can be utilized for the purpose of developing representative human models for chemotherapeutic drug testing. Even the simplest spheroid tumor models showed several advantages over 2D cell culture systems. For instance, Grandis *et al* used 3D multicellular DU-145 prostate cancer cell line based spheroids that could effectively simulate the prostate tumor microenvironment to evaluate the cytotoxic effects of ruthenium complexes [43]. The format chosen and its alterations depend on the purpose of the model. For example, for the preliminary screening of an anticancer agent, a 2D cell culture system composed of a single type of cells would be sufficient. If the goal is to test the efficacy of a chemotherapeutic agent in a heterogeneous tumor environment, a spheroid model composed of cancer cells, cancer stem cells, circulating tumor cells (CTCs) and different stromal cells should be incorporated. However, to get a clear understanding of drug penetration, anti-metastatic effect and clearance, a 3D model composed of biopolymeric scaffolds, cancer cells and multiple types of stromal cells with vascular channels needs to be developed [44].

In pursue of personalized drug screening, multicellular organoids can be generated using different types of patient-derived cells isolated from biopsy tissues in a co-culture system capable of creating spheroids. The major advantage of these spheroid systems is that the screening can be performed with very small quantities of chemotherapeutic candidates [45], [46], [47]. However, spheroid models cannot completely recapitulate the cellular and microenvironmental heterogeneity of physiological tumor tissue where spatiotemporal variations in nutrients supplement, cell proliferation and oxygenation often occur. A number of reports show that significant difference between the 2D and 3D models in many of the major physiological features of cancer cell culture, such as expression of genes and proteins, cell migration and cell proliferation are very important factors in personalized chemotherapeutic screening [30]. Lastly, 3D cancer models can be maintained in bioreactors where the cell culture parameters can be precisely regulated and monitored in real-time, which is not possible in static 2D cultures [48]. Therefore, creating heterogenous and physiologically related 3D cancer tissue constructs is expected to expand our understanding of the effects and mechanisms of chemotherapeutic agents on the cancer growth, progression, metastasis and therapy response [49]. For high-throughput screening or tissue engineering, researchers tend to prefer automated approaches such as those employing 3D bioprinting. With the help of bioprinting, it is possible to generate cell laden cancer tissue constructs that can recapitulate the features of various types of cancers, and used for drug screening and preclinical testing [50]. 3D tumor constructs generated by 3D bioprinting approaches have expressed characteristics of *in vivo* tumor tissues, such as high growth rates of cancer cells, aggressive invasiveness, angiogenesis, metastasis, high resistance to anticancer drugs [18]. Moreover, 3D bioprinted tumor constructs can supplement animal xenograft models because they maintain cancer–stromal cell interactions.

## 4 An overview of 3D bioprinting

3D bioprinting is an innovative biomanufacturing platform, which allows the deposition of living cells, signaling molecules and biomaterials using computer-aided design (CAD) to generate tissue engineered constructs with highly controlled tissue architecture [51,52]. Bioprinting technology can create graded macroscale architectures mimicking the ECM, thereby enhancing the attachment and proliferation of different cell types, simultaneously. 3D bioprinted constructs can effectively mimic the tumor microenvironment. Moreover, spatial control of matrix properties, ability to integrate perfusable vascular networks, automation and high-throughput testing abilities for determining metabolic, and toxicological properties are imperative features of bioprinted cancer models [18].

Utilizing bioprinting technology, many complex internal tissue structures including channels and stroma with varying dimensions can be generated [53]. Consequently, it is also possible to produce different layers of cells including normal tissue specific cells, connective tissues and cancer cells [54]. Some of the important factors influencing bioprinted cancer constructs are physicochemical properties of biomaterials, their concentration/viscosity, features of cells, cell concentration, printing process, flow, printing time, extrusion pressure, crosslinking techniques, post-processing, and cell culture conditions [55]. Many of these parameters need to be optimized to unveil the full potential of this technology [56]. At present, natural polymers like alginate [57], gelatin [58], collagen, fibrin and hyaluronic acid are widely used in bioprinting [59]. Similarly, many combinations of synthetic and naturally derived hydrogels (E.g. Gelatin methacryloyl) are being used within bioinks to print robust tissue constructs [60,61]. Crosslinking is an essential step in bioprinting enabling production of a bioprinted construct that is stable under physiological conditions [62]. Stabilization and strengthening of bioprinted constructs can be achieved through physical or chemical crosslinking methods [63]. Chemical crosslinking methods can be based on enzymes (e.g., mushroom tyrosinase for gelatin) [64], tannic acid (for collagen crosslinking), and divalent cations such as calcium ions (for alginate) [65]. Physical crosslinking approaches including ultraviolet (UV) -treatment (e.g., for gelatin methacryloyl (GelMA)) are also being used for stabilizing the cell-laden bioprinted constructs [66].

**Fig. 2:**
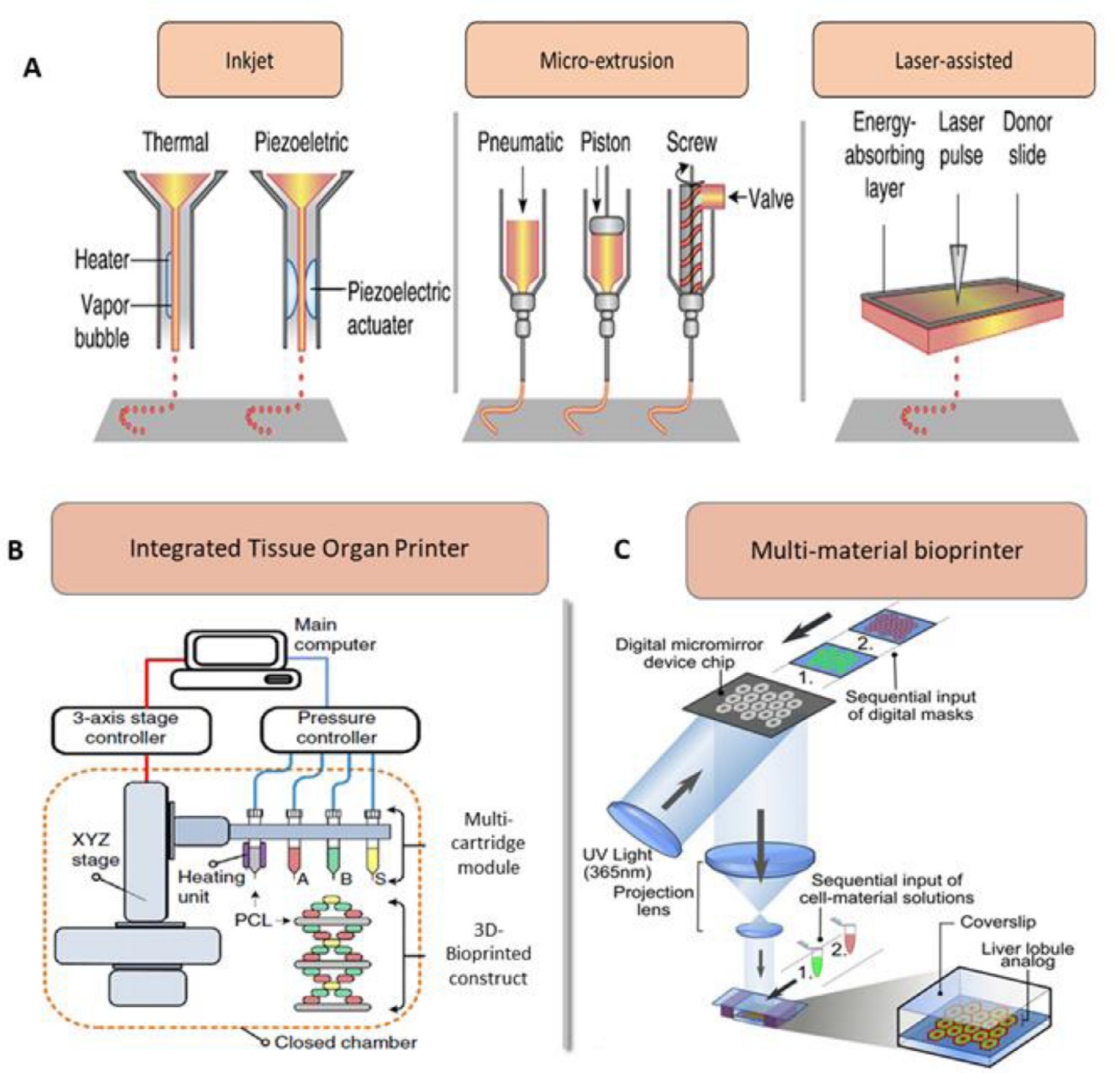
Various methods of bioprinting used in the development of bioengineered cancer tissues. (A) Components of inkjet, microextrusion based and laser-assisted bioprinters. **(B)** A scheme showing the multi-cartridge module based Integrated Tissue Organ Printer (ITOP) system consisting of stage/controller, dispensing module with pressure controller and a closed chamber. **(C)** Schematic diagram of a two-step 3D bioprinting method in which hiPSC-HPCs were patterned by the first digital mask followed by the patterning of supporting cells using a second digital mask. Figure A is reproduced from [52] with the permission of Springer Nature. Figure B is reproduced from [76] with the permission of Springer Nature. Figure C is reproduced from [77] with the permission of PNAS.

Based on instrumentation approaches, bioprinting approaches can be classified into droplet, extrusion and laser-based bioprinting depending on their deposition mechanism [67] (Fig. 2-A). In droplet-based bioprinting approach, different energy sources including sound, temperature, and electricity are used to produce bioink droplets in a high-throughput manner [68,69]. Extrusion based bioprinters consist of a fluid dispensing system for bioink extrusion and an automated robotic system for accurately depositing bioink on the platform according to the design. Recently, a rational integration of microfluidic systems with extrusion printing was adopted to attain rapid deposition of bioink [70] (Fig. 2-B). It is reasonable that the extrusion-related stress may alter cell viability [71,72]. Laser-based bioprinting facilitates high-precision patterning or fabrication of tissue constructs with the help of laser energy. In laser-assisted bioprinting (otherwise called biological laser printing, LAB), to generate vaporizing sacrificial layer, laser energy is used in the system to push a cargo to a receiving substrate (in nozzle-free bioprinting) [73]. Other methods used for bioprinting include mask patterning with similar functionalities to silicon chip patterning process (Fig. 2-C). Laser direct-write (LDW) bioprinting technique is an excellent technique for precisely encapsulating multiple types of cells within microbeads [74]. These can be used to produce embryoid bodies (EBs) and MCTSs with desired manipulation of size and shape thus showing tremendous potential as an innovative technique for generating bioprinted tumor constructs [75].

### 4.1 Utilization of cancer cells and stromal cells in 3D bioprinted cancer models

For generating a personalized bioprinted construct, patient-specific cells and their microenvironment need to be recapitulated in the printed construct. The success of the fabricated construct is highly dependent on the selection of appropriate cells for bioprinting. Multiple types of cancer cells including primary cancer cells, CTCs, and stromal cells including fibroblasts, endothelial cells and stem cells can be used for printing personalized tumor construct. Of note, stem cell-like phenotype was observed in personalized bioprinted tumor construct using a gelatin-alginate-Matrigel bioink containing patient derived intrahepatic cholangiocarcinoma cells [78]. This construct also showed the expression of several cancer-associated biomarkers, cancer stem cell markers, and the evidence of epithelial to mesenchymal transition (EMT). These studies could further pave the way for bioprinting of multicellular patterns with patients own heterogenous cancer cells as a platform for testing chemotherapeutic agents and hence the development of personalized therapies [79].

Apart from cancer-associated cells, a bioprinted tumor construct should contain stromal cells to mimic a natural tumor microenvironment of human tumors where the cancer cells are encapsulated within different types of healthy cells. Selection of these normal cells in bioinks should be based on the tumor characteristics and bioengineered accordingly. For instance, a brain tumor construct should be based on patient-derived tumor cells of interest mixed with surrounding healthy brain cells including neurons, glial cells, astrocytes, and microvascular endothelial cells [80,81]. However, a breast cancer construct should be designed in such a way that patient-derived breast cancer cells are surrounded by mammary epithelial cells, adipocytes, fibroblasts, and endothelial cells [82,83]. This approach facilitates recapitulation of the tumor type as well as the target tissue.

### 4.2 Bioinks for cancer bioprinting

Bioinks are the building blocks of bioprinted constructs, which are generally composed of a suitable hydrogel, multiple types of cells, nutrients, and growth factors. The hydrogels used in bioinks should be biocompatible, and their gelation should be easily controllable [67]. Moreover, these biopolymeric hydrogels should mimic structural, physicochemical, and biological properties of ECM to provide representative tumor microenvironment within the constructs. Biopolymers such as alginate, collagen, and agarose are widely used matrices for bioink due to their low cytotoxicity, high water content and innate biocompatibility favoring cell adhesion, proliferation, differentiation, and maturation [84,85]. Viscosity of bioinks is a critical factor that determine the printability and quality of printed tumor constructs, which is related to the chemical components and concentration of biopolymer used in bioinks [86,87]. Crosslinkability of biopolymers used in bioink is another important factor that can impact the reliability of printed construct. Sodium alginate with Ca^2+^ or other divalent cations are commonly used due to fast gelation and minimum adverse effects on cells [88], [89], [90]. In order to improve cell proliferation in bioprinted construct, alginate can be combined with other bioactive hydrogels like collagen [91,92]. Photocrosslinkable hydrogels are another advancement in bioprinting. A methacrylated form of gelatin (GelMA), is one of the most successful photocrosslinkable hydrogel used in bioprinting [93], [94], [95], [96]. In addition to the importance of selecting adequate bioinks and crosslinking methods for generation of viable bioprinted cancer models, the methodology of cell integration is also critical, which requires preparation of cancer and stromal bioinks separately.

Cancer bioink: Cancer bioinks are composed of patient-derived cancer cells (e.g. primary cancer cells, cancer stem cells, CTCs, cancer-associated fibroblasts, CAF), suitable biopolymer (e.g. GelMA, alginate, hyaluronic acid and collagen), growth factors (EGF, FGF) and other nutrients (e.g. cell culture medium). For this purpose, a non-globular protein, fibrin, important for blood clotting [97], and a gelatinous ECM protein secreted by the mouse sarcoma cells, Matrigel [98], are widely used. These hydrogel matrices provide an optimum microenvironment for cancer cell proliferation and tumorigenesis to generate a tumor tissue with relatively similar structural as well as functional features to *in vivo* tumors [99,100]. Schiele et al. reported successful encapsulation of neural stem cells, pulmonary artery endothelial cells, myoblasts, dermal fibroblasts, breast cancer cells within Matrigel™-based constructs [101]. Recent studies showed that the presence of CTCs, capable of disseminating cancer cells to distant locations are the major contributors to the development of metastatic cancer [102]. Hence, it is important to incorporate these cells within cancer bioinks, facilitating the investigations into anti-metastatic effects of chemotherapeutic agents [103]. Similarly, cancer stem cells consist of a small percentage of the tumors and are highly tumorigenic and treatment resistant [6,104,105], therefore incorporating these cell types within tumor construct will provide a better insight into treatment response. In order to develop personalized tumor constructs for precision drug screening, these heterogeneous population of cancer cells should be present within bioprinted cancer constructs before chemotherapeutic combinations of treatments are tested [54]. However, the most challenging aspects of creating heterogenous cancer constructs include: prompt isolation of sufficient number of cells, maintenance of these heterogeneous group of primary cells, subsequent bioprinting and rapid testing with drug candidates that will enable selection of the most appropriate chemotherapy.

Stromal bioinks: In addition to primary cancer cells, tumor tissue is also composed of heterogeneous group of healthy stromal cells including endothelial cells, mesenchymal/hematopoietic stem cells, fibroblasts and other tissue specific cells [106,107]. Similar to cancer bioinks, both natural and synthetic polymers have been used for creating stromal bioinks including hydrogel matrices [108]. In order to recapitulate the microenvironment of physiological stroma of tumor tissue within bioprinted constructs, preferably, patient-derived primary cells from the tumor tissue should be isolated, cultured and mixed with the hydrogel to generate the bioink rather than using biobanked primary cells and cell line. Nevertheless, this is not always feasible, hence the most suitable biobanked stromal cells should be selected for the tumor type. *In vivo*, endothelial cells or endothelial progenitor cells are recruited by tumor immune cells and others to initiate tumor angiogenesis through an array of secreted signaling molecules. Generally, tumor blood vessels have special features including extensive branching and loose intercellular junctions making them leakier than normal blood vessels. Thus, incorporating endothelial or endothelial progenitor cells along with key angiogenic factors such as VEGF or EGF in stromal bioink is crucial for recapitulating tumor growth [109]. Nevertheless, generating vascularize tumor construct still remains a challenge in bioengineering.

### 4.3 3D bioprinting, *in vitro* maturation and characterization of bioprinted construct

Briefly, the process of bioprinting starts with the CAD design of the structural architecture of the specific tumor tissue. The bioink can have multiple types of patient-derived malignant and healthy cells (cancer and stromal bioinks). The cells are mixed with other components of bioink (biopolymers, media and growth factors) at the ratio required to mimic the *in vivo* tumor architecture and microenvironment [110]. The bioinks are then extruded to print the construct as per CAD design. Recent approaches such as immersion printing, are beneficial in developing tumor constructs in multi-well plates therefore increasing the throughput of personalized drug screening [111]. Integrated microscale continuous optical 3D bioprinting can also be used to generate tumor constructs in multi-well plate formats [112]. These techniques facilitate the development of constructs with varying spatial geometries with the added advantage of reproducibility. Once the complete model is printed layer by layer, the construct is exposed to final crosslinking (photocrosslinking or ionic crosslinking depending upon the hydrogel composition) and then maintained in a suitable culturing medium for maturation [113]. Biochemical gradients in tumor tissues greatly impact cellular processes such as cell adhesion, cell migration, cell proliferation, differentiation and angiogenesis. To provide a chemical gradient to simulate the proper maturation of bioprinted construct, multiple active agents can be loaded as multiple layers with varying layer spacing [114].

*In vitro* tumorigenesis facilitated by cell division, proliferation and differentiation during maturation of bioprinted constructs is a critical step in the post‐printing stage on cancer constructs. Achieving rapid maturation of bioprinted construct is key especially when these are utilized for screening of personalized anti-cancer therapies. This is time sensitive and requires identification of the most suitable chemotherapy regimen that could be administered to the cancer patients. Nevertheless, accelerated tissue maturation is challenging and further research and optimization is required to progress the development of personalized bioprinted tumor constructs for precision chemotherapeutic screening. Past studies indicated that tissue maturation can be achieved in static culture systems to some extent [115]. Recently it has been reported that incubation of tissue spheroids for different time periods affected tissue fusion kinetics [116], which is an important factor to consider with maturation of bioprinted cancer constructs. Incubation of tissue spheroids in hanging drop cultures for long duration appears to stimulate tissue cohesion and maturation along with improved accumulation of ECM molecules [117].

Several physicomechanical measurements and biological assays are required to properly characterize bioprinted cancer constructs before using these for personalized drug testing. Stiffness of the construct can be measured by nanoindentation using an Atomic Force Microscope (AFM) [20]. Scanning electron microscopy of fixed and dried construct can be performed to characterize the topographical features of the bioprinted construct. It is also key to verify the viability of cells present within the bioprinted construct promptly after bioprinting and throughout the course of *in vitro* maturation and maintenance. Viability of different types of cells within the constructs can be tested by calcein-AM based staining which can label live and dead cells by staining live cells green. Depending upon the 3D model, different types of immunohistochemistry or immunofluorescence assays can be performed to understand the expression of various proteins important in the development and maintenance of cancer progression, synthesis of ECM components and membrane proteins [52]. Once the bioink composition, process of fabrication, and maturation conditions, are optimized, developed tumor construct can be used for drug testing.

Personalized bioprinted tumor constructs after maturation can be used to test an array of potential chemotherapeutic agents. In static culture systems, the chemotherapeutic agents can be administered to a tumor construct within the cell culture medium to determine the effects on cell viability and tumor growth reduction. Bioprinting can be performed in multi-well plates, generating large numbers of homogeneous organoids thus increasing the throughput of screening [111,118]. In a more realistic bioprinted tumor constructs, chemotherapeutic agents can be administered through built-in micro- channels that mimic tumor vasculature [119]. Tumor constructs can also be printed on microfluidic chips or integrated with similar platforms to automate the screening assay and perform the analysis of a number of drugs simultaneously as well as determine the response and mechanism in real-time [120,121].

## 5 Cancer-specific bioprinted models for precision chemotherapy screening

### 5.1 Bioprinted breast cancer models

**Fig. 3:**
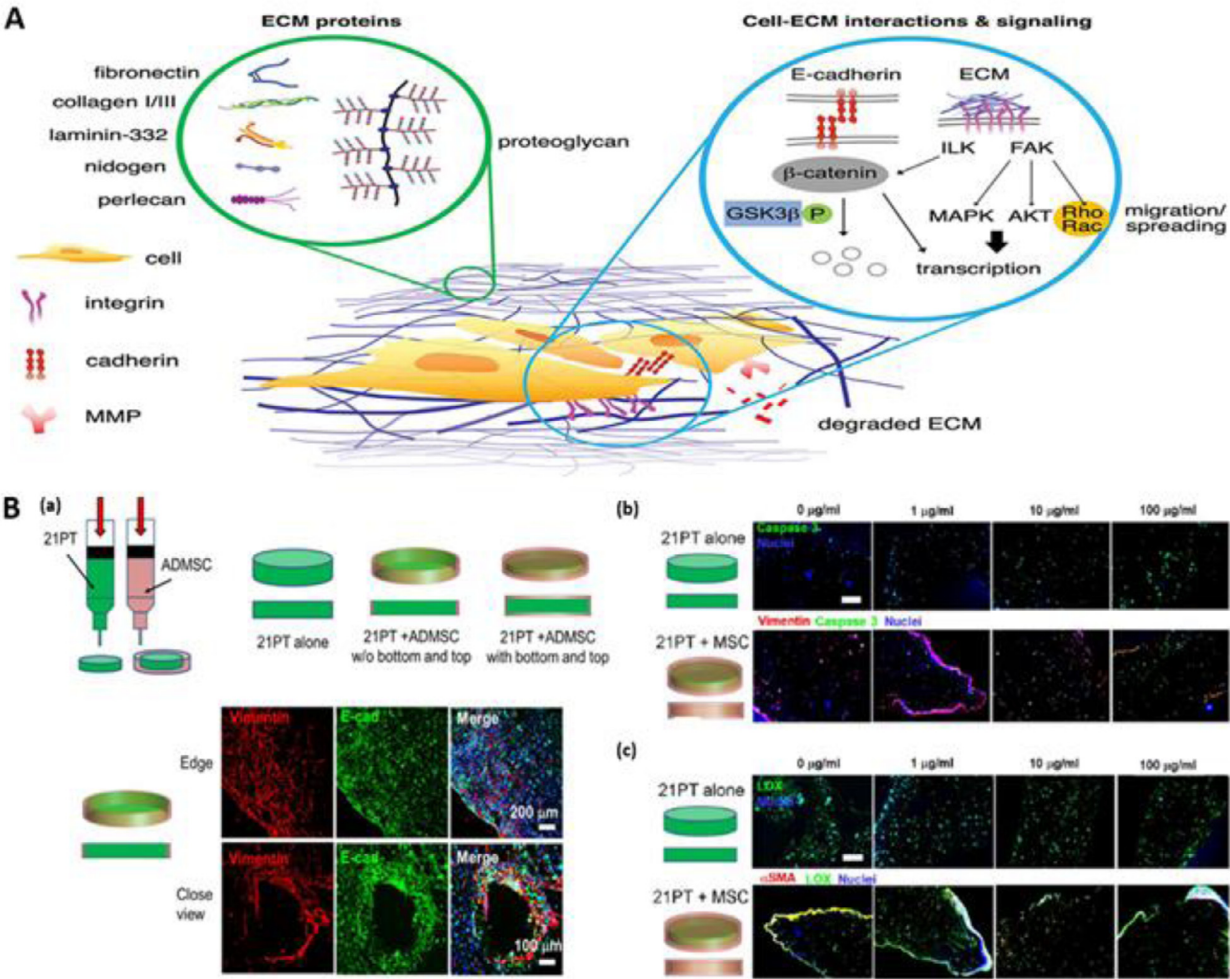
**Bioprinted breast cancer models. A.** The important components within breast cancer microenvironment including interstitial matrix and the basement membrane proteins (left circle). The signaling from the ECM proteins is propagated via multiple signaling pathways, both simultaneously and independently (right circle). **B.** 3D bioprinted breast cancer model with breast cancer cells (21PT cells) in the middle and surrounding Adipose-Derived Mesenchymal Stem Cells (ADMSCs). **(a)** Scheme of bioprinting setup that uses two types of cell laden bioinks. Representative immunofluorescent staining for vimentin (red), E-cadherin (E-cad, green), and nuclei (blue) of 21PT and ADMSC within bioprinted constructs. **(b)** Representative immunofluorescent staining of vimentin (red), caspase 3 (green), and nuclei (blue) of 21PT and ADMSC cells after 21-day culture and with the addition of different concentration of doxorubicin (DOX) for another 3-day culture. **(c)** Representative immunofluorescent staining for aSMA (red), LOX (green), and nuclei (blue) of 21PT and ADMSC within bioprinted constructs after 21-day culture and with the addition of different concentrations of DOX for another 3-day culture. Figure A is reproduced from Ref. [109] with creative commons attribution (CC BY) license. Figure B is reproduced from Ref. [20] with the permission of American Chemical Society.

Breast cancer is the most frequently diagnosed cancer in women worldwide, with approximately 2.09 million new cases and 627,000 deaths in 2018, estimated by the WHO [122]. Most cancer-related deaths result from breast cancer metastasis to vital organs indicating the importance of early diagnosis and treatment [123]. Along with malignant cells, stromal cells of breast cancer also greatly influence the progression of tumor by altering the cells phenotypes, secreting signaling molecules and reorganizing themselves to support tumor invasion [109] (Fig. 3-A). Moreover, immune cells within the stroma also play important roles in modifying the tumor microenvironment [124]. For example, inflammation and angiogenesis are two major factors influencing the ECM matrix elements of tumor microenvironments [125]. Thus, reliable breast cancer models should be based on patient-derived cancer cells and surrounding healthy cells (e.g. fibroblast, immune, epithelial and endothelial cells) to recapitulate a functional *in vivo* tumor microenvironment. Initial studies in this field were focused on the development of bioprinted breast cancer spheroids using widely available breast cancer cell lines and synthetic hydrogels [126]. Bioprinting based on cellular spheroid construction was demonstrated by Ling et al. using MCF-7 breast cancer cells and gelatin hydrogel showing the advantage of uniform cell distribution in spheroids and the potential for anticancer drug screening [127]. Swaminathan et al. developed bioprinted construct using pre-formed spheroids of human breast epithelial cell lines [128]. They also developed bioprinted constructs using individual cell loaded bioinks for comparison. Pre-formed breast spheroids maintained their architecture, viability and cellular function after bioprinting. However, individual breast cells only spontaneously formed spheroids in Matrigel-based bioink. Moreover, spheroids-based constructs showed higher resistance to paclitaxel than individual cell based bioprinted constructs, which could be linked to enrichment in cancer stem cells within spheroids. Mollica et al. reported the successful development of bioprinted organoids and tumoroids using a 3D hydrogel comprised ECM proteins [129]. It was noted that co-culturing the breast cancer cells (MDA-MB-231) with osteoblasts or mesenchymal stem cells (MSCs) in bioprinted constructs improved the secretion of VEGF compared to mono-culture of cancer cells [130]. Moreover, the breast cancer migration potential was improved in newly developed 3D bioprinted models compared to 2D tumor models. Additionally, co-culturing of [131] adipose- [20] or bone marrow- derived [132] MSCs with cancer cells enhanced the spheroid colony formation within 3D bioprinted constructs (Fig. 3-B). Recent studies demonstrated that bioprinting of the cells in a co-culture with adipocytes resulted in morphological changes and differences in cell localization within printed structures [131]. Integration of vascular networks within bioprinted constructs would be a closer step towards the development of physiologically relevant engineered cancer tissues. A proof-of-concept of bioprinted vascularized tumor constructs was provided by Cui et al in their interesting research on 3D printed breast-bone metastatic model [133]. Liu et al. developed a human breast tumor model with physiologically relevant embedded lymphatic vessels by sacrificial bioprinting [134]. This offers promise for developing and screening personalized tumor anti-lymphangiogenic therapies in the future. Recent investigation suggested that the gene alterations during bioprinting are evident demonstrated by changes in the expression of LUCAT1, IL6, CCL26, and NRN1L genes and phosphorylation of critical oncogenic drug resistance pathways in breast cancer cells in thermal bioprinted constructs [135]. Understanding such changes at the molecular level in bioprinted constructs would help to develop models that mimic human tumor tissues. A proof-of-concept study utilizing bioprinted breast cancer constructs was reported by Li et al. [136]. Using a hydroxyethyl cellulose/alginate/gelatin hydrogel and MCF-7-based spheroid bioprinted construct, the anti-breast cancer activity of phosphoramidates (13 amino acid-containing flavone) was examined and the results indicated that alanine structure induced a stronger drug resistance than phenylalanine in the MCF-7 cells. The development of advanced screening and drug testing platforms for precision medicine applications has progressed significantly based on the advancements in 3D co-cultured breast cancer models [137].

### 5.2 Bioprinted brain tumor models

**Fig. 4:**
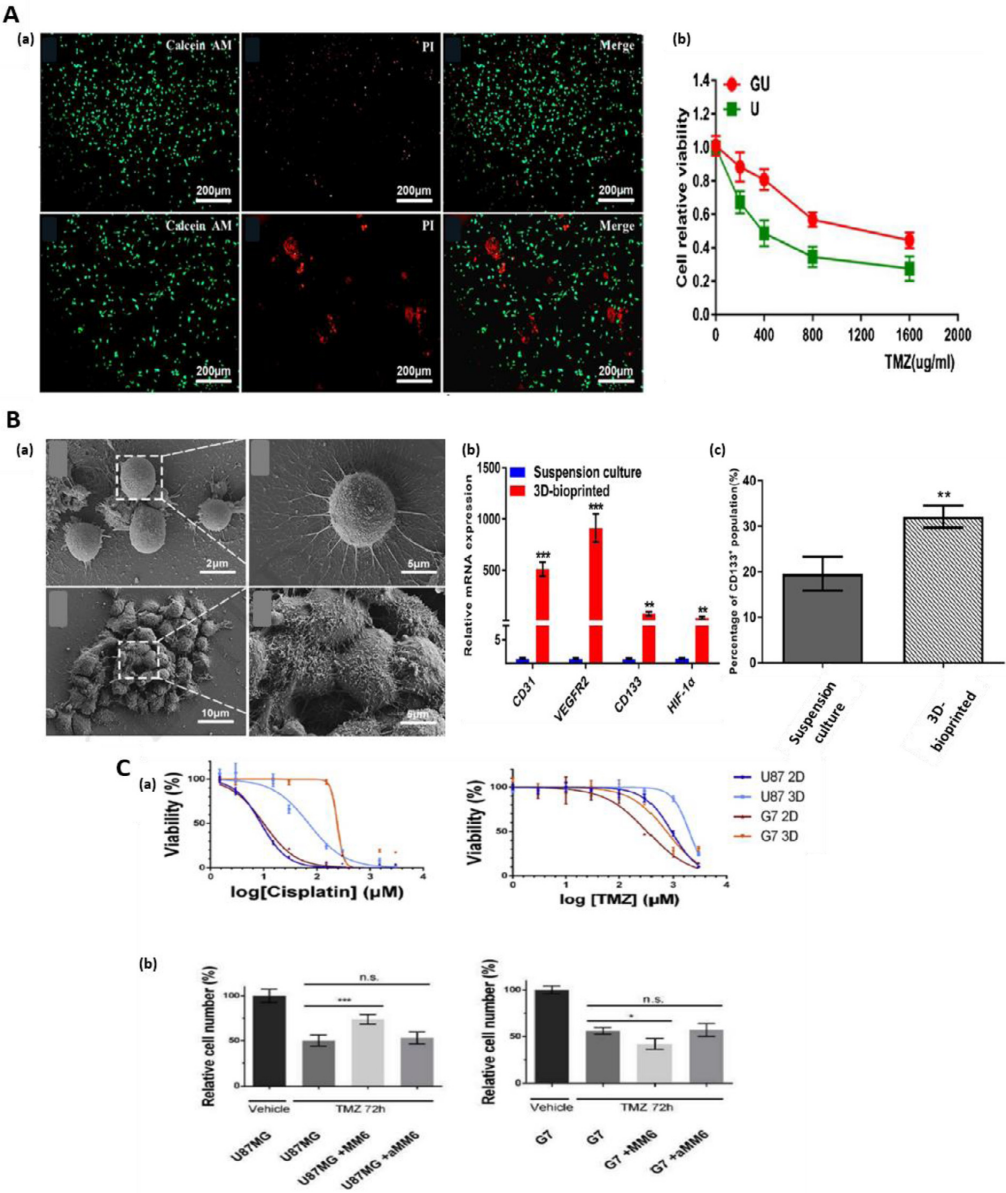
Bioprinted brain tumor models: A. (a) Microscopic images of U118 cells collected from hydrogel fibers and U hydrogel microfibers after temozolomide (TMZ) treatment, dead cells (red) accumulated into clusters. (b) Cell viability after treatment with TMZ. B. (a) SEM images of cell clusters from suspension culture and from 3D bioprinted constructs. (b) The expression of angiogenesis-related genes (CD31, VEGFR2, HIF-1α and CD133) in 3D bioprinted tumor constructs and suspension culture. The percentage of CD133+ cells in 3D bioprinted scaffolds in comparison to suspension culture. C. (a) Drug dose response of glioblastoma cells in 3D bioprinted models. U87MG or G7 cells were cultured in 2D or 3D printed in RGDS-Alginate after treatment with increasing concentrations of either cisplatin or temozolomide (TMZ) for 72 hours. (b) U87MG-EGFP and G7- EGFP, were 3D printed either alone or in co-culture with either MM6 cells, aMM6 cells or primary human microglia. Fig. A is reproduced from Ref. [144] with the permission of Elsevier. Figure B is reproduced from Ref. [35] with the permission of Elsevier. Fig. C is reproduced from Ref. [151] with the permission of Elsevier.

About 10–40% of brain tumors are metastatic at the time of diagnosis and hence when developing *in vitro* models of brain tumors, this should be taken into the account [138]. Bioprinted brain tumor constructs for drug screening/personalized treatment regimens can be particularly beneficial for glioblastoma (GBM), the brain tumor type that shows resistance to a number of chemotherapeutic agents [139,140]. In addition to the overall poor response of GBM to chemotherapy, heterogeneity within these tumors and inter-patient variations in treatment response [141] are also having negative impact on patient outcomes. This unmet clinical need could be addressed through the development of personalized therapeutic approaches and novel therapies that are tested on patient-derived bioprinted constructs with a potential to improve patient survival rates [142,143]. Initial studies that focused on the development of brain tissues using 3D bioprinting technique with different types of brain cancer and healthy cells including stem cells provided a proof-of-concept for the generation of bioprinted brain tumor constructs constituting heterogeneous group of cells [21]. These models can reliably recapitulate *in vivo* tumor microenvironment and represent promising personalized GBM platforms for chemotherapeutic screening. The results from a study investigating temozolomide treatment for GDM, showed that 3D tumor models containing glioma stem cells were more resistant to temozolomide treatment compared to 2D monolayer, thus likely recapitulating physiological tumor response more closely [142]. Wang et al. developed bioprinted GBM tumor models using glioma cell line U118 within the core of the construct and glioma stem cell, GSC23, on the shell of hydrogel microfibers by coaxial extrusion bioprinting technique [144] (Fig. 4-A). Developed constructs were able to better mimic glioma microenvironment, which was supported by observed resistance to chemotherapeutic agents and higher expressions of tumor invasiveness markers including vascular endothelial growth factor receptor-2 (VEGFR2), matrix metalloproteinases (MMP2, MMP9) and O6-methylguanine-DNA methyltransferase (MGMT). Another interesting study from the same group indicated that 3D bioprinted glioma stem cell-containing constructs showed the expression of tumor angiogenesis-associated genes and higher vascularization potential [35] (Fig. 4-B). Yi et al. demonstrated that 3D bioprinted GBM constructs composed of patient-derived cancer cells, vascular endothelial cells and brain tissue-derived ECM into concentric-ring structure are reliable platforms of GBM microenvironment in terms of structural, biochemical and biophysical characteristics [145]. A key feature of GBM is high degree of vascularization [146], and this uncontrolled tumor vascularization aids in rapid progression and invasion of this tumor [147]. Thus, chemotherapeutic approaches targeting GBM are focused on the treatments capable of blocking key mechanisms of tumor angiogenesis [148]. Hence, to study the potential of chemotherapeutic agents in GBM, it is key to establish a well-connected vasculature in bioprinted cancer construct [149]. Moreover, it has been reported that a subtype of glioma stem cells within the GBM tumor can differentiate into endothelial cells promoting angiogenesis [150]. Use of such tumor angiogenic cells in bioinks can generate a vascular network within the construct. This was replicated by stimulating angiogenesis through cultured GSCs secreted factors present in the cell medium [32,35]. A recent study managed to recapitulate the heterogeneous glioblastoma tumor microenvironment in bioprinted constructs using an array of cancer and stromal cells including MM6 monocyte/macrophages, U87MG glioblastoma cells, glioblastoma stem cells, microglia and glioma associated stromal cells (GASCs) providing a more realistic chemotherapeutic response [151] (Fig. 4-C). In an interesting study, a 3D‐bioprinted mini‐brain incorporated with Glioblastoma‐associated macrophages (GAMs) and glioblastoma tumor cells was created to study the phenotypic alteration in both cancer cells and macrophages using a a two-step bioprinting process [21]. This study clearly demonstrated that this 3D‐bioprinted tumor model is not only able to mimic intact GBM, but also applicable to other types of cancers and can be used as a tool to improve the understanding of tumor biology as well as for testing chemotherapeutic drug efficacy. An immersion bioprinting technique has also been practiced in patient-derived GBM and sarcoma biospecimens, which can mitigate the limitations of patient derived tumor organoids(PTO) for drug screening [111].

### 5.3 Bioprinted skin cancer models

Skin cancer or melanoma is one of the most common forms of cancers especially in Caucasian people consisting of different cancer types including non-melanoma skin cancer (NMSC), malignant melanomas (MM), Merkel cell carcinomas (MCC), basal cell carcinomas (BCC) and cutaneous squamous cell carcinomas (cSCC), that affects over 300,000 people annually across the globe [152]. Laser-assisted bioprinting technique was used to develop a first batch of bioprinted artificial skin constructs in a collagen matrix embedding multiple layers of fibroblasts and keratinocytes [153]. In order to develop adequate vascularization, micro-channels were generated from alginate bioinks using a 3D bioprinter [154]. The 3D bioprinted modified constructs were able to form perfusable vasculature within dermal and epidermal skin layers [155]. Similarly, spheroids based on primary melanoma cells or cell lines are also good candidates for investigating the effectiveness and mechanism of personalized treatments in melanoma. Encapsulation of stroma cells within 3D constructs mimic accurately tumor microenvironment as these cells secrete growth factors and ECM components needed for the tumor growth and migration potential. Melanoma co-culture models frequently use fibroblasts, immune cells, and endothelial cells, in combination with melanoma cells. The co-culture models can be seeded both following cancer MCTS formation or simultaneously with tumor cells in non‐adherent conditions [156,157]. In another important study, 3D human skin equivalents (HSE) and melanoma skin equivalent (MSE) from human de‐epidermized dermis (DED), keratinocytes and fibroblasts were utilized for the study of vertical migration and invasion of myeloma cells. The developed skin constructs resemble human skin with pronounced stratification of dermis and epidermis and a basement membrane. Interestingly, the keratinocytes cells confined themselves to epidermis and fibroblasts differentiated into dermis within the HSE and MSE [158,159]. A combination of TRAIL (tumor necrosis factor-related apoptosis-inducing ligand) and ultraviolet-B radiation (UVB) or cisplatin led to the development of an organotypic human skin-melanoma model with a potential to be used as a platform for precision medicine approach and tailor treatment regimens [160]. Before the development of 3D HSEs, Hill and colleagues developed a representative skin model based on the human fibroblasts laden within an inert porous scaffold where the myeloma cells move radially and vertically during tumor progression [161]. As indicated in a preliminary study, 3D bioprinting techniques can be used to fabricate skin constructs of varying pathophysiological features, either as non-vascularized or vascularized forms, and in a multi-well platform to enable drug screening [162]. Recent advances like immersion bioprinting of patient derived sarcoma and its use in drug screening will be a game changer in personalized cancer therapy [111].

### 5.4 Bioprinted colorectal cancer models

Colorectal cancer (bowel cancer, colon cancer, or rectal cancer) is associated with one of the poorest survival rates and it is often diagnosed at the metastatic stage [163]. Timely identification and treatment of colorectal cancers can prevent the spread of tumor to surrounding areas and metastases to distal organs. Colorectal cancer mostly arises from dysplastic adenomatous polyps [164]. Adenocarcinoma is the most common type of colorectal cancer comprising around 95% of the cases [164]. Intestinal spheroid models and organoid models with human colon adenocarcinoma, adenoma, and Barrett's epithelium were developed showing promising cancer models [165]. The stem cell-like properties of these organoids can be propagated and stored in a biobank for future application in pre-clinical and clinical research [166]. Recent advances in the techniques of 3D bioprinting have enabled researchers and clinicians to develop 3D colorectal models that mimic the exact mechanisms of cancer initiation, growth and invasion and focus on personalized drug treatments. In colorectal carcinoma, the success of personalized drug treatment is reliant upon accurate diagnosis based on histopathological and imaging assessments of the patients, the likelihood of tumor recurrence following tumor debulking and the anticipated response to adjuvant systemic chemotherapy. It was reported that developing 3D bioprinted tumor models by encapsulating colorectal cells within alginate polymer bioinks reduced the hypoxic regions compared to spheroid cultures, thereby showing better suitability for precision medicine screening application [167,168]. Furthermore, recent advances utilizing induced human pluripotent stem cells (iPSCs) showed this approach could generate intestinal organoids for the development of patient specific *in vitro* models and personalized drug screening [169], [170], [171].

### 5.5 Bioprinted cervical cancer models

Cervical cancer continues to be the leading cause of death in women despite recently introduced vaccination and screening, hence with an urgent need for improvement in personalized screening, and treatment efforts [172]. The potential precursor of cervical cancer is cervical intraepithelial neoplasia. Cervical cancer is divided into two main subtypes namely squamous cell carcinoma and adenocarcinoma that account for 70% and 25% cervical cancer cases, respectively [173]. Squamous cell carcinoma develops within the thin flat cell lining of cervix hence, known as squamous cell carcinoma, which later projects into vagina. On the other side, adenocarcinoma is found in cervical canal line close to column shaped glandular cells. For tumorigenesis study and anti-cancer drug screening, bioprinted models of cervical cancer have showed substantial improvement and utilization in mimicking the physiological environment. In this context, HeLa cells were loaded in a hydrogel scaffold comprised of gelatin, alginate, and fibrinogen myofibrils to bioprint 2D and 3D tumor construct of cervical cancers [31]. HeLa cells developed into 3D spheroids in 3D cultures while monolayer cell sheets were formed in 2D culture. In addition, HeLa cells showed a marked increase in the expression of MMP proteins in 3D bioprinted models, which are capable of degrading ECM components and these 3D models were more sensitive to paclitaxel treatment. The findings of this study demonstrated the benefits of these 3D bioprinted *in vitro* cervical cancer models for investigating mechanisms and treatment options for preventing cervical cancer progression. It was observed that HeLa cells migrated to different extents depending on the channel diameter and no effects were reported on fibroblast migration [174]. Transforming Growth Factor-β (TGF-*β*) induced (EMT) in an advanced bioprinted cervical tumor model shows promising results for developing future therapeutic strategies towards preventing or treating cervical tumor metastasis. A HeLa/hydrogel grid construct comprising alginate, gelatin, Matrigel and HeLa cells showed rapid proliferation, formation of spheroids and displayed tumorigenic feature in the 3D-printed construct. The down-regulation of epithelial marker, E-cadherin, and up-regulation of mesenchymal markers including snail, vimentin and N-cadherin, were reported in the 3D-printed model with the supplementation of TGF-*β*. Furthermore, the TGF-*β* induced EMT was inhibited following treatment with disulfiram and EMT pathway inhibitor C19 in a dose-dependent manner [175]. Despite significant developments in cervical cancer prevention, diagnosis, and treatment, there are still disparities in patient outcomes, hence further research efforts are required for implementation of precision medicine, which may be addressed through utilization of patient-specific bioprinted cervical cancer constructs for drug screening and mechanistic insight [176].

### 5.6 Bioprinted pancreatic cancer models

Pancreatic adenocarcinoma is the second most lethal cancer in the world. Advance stages of pancreatic cancer have very poor prognosis with a five-year survival rate ∼2–5% cases [177] . There are different types of pancreatic cancers with pancreatic ductal adenocarcinoma (PDAC) being the most common (95%) type [178]. Within the pancreatic carcinomas, pancreatic cancer cells are surrounded by a scar-like dense fibrous tissue, which acts as a barrier containing protective proteins that abrogates body's own anti-cancer defense mechanisms and the effectiveness of drug treatments. Therefore, there is an unmet clinical need for representative models that could help our understanding and provide insight into the effectiveness of different treatment regimens, particularly for PDAC. These models should mimic *in vivo* tumors and microenvironment and include strategies for recapitulating surrounding fibrous tissue containing barrier proteins [179]. It is well documented that stromal cells in pancreatic cancer account for more than 80% of the total tumor volume and secrete a number of signaling molecules including fibroblast growth factor (FGF), epidermal growth factor (EGF), VEGF, TGFβ and insulin-like growth factor Ⅰ (IGF-Ⅰ) as well as matrix metalloproteinases (MMPS) [180]. Overexpression of these signaling factors fuels the progression of pancreatic cancer by enhancing cell proliferation, migration and invasion [181,182]. Physiologically pertinent 3D organotypic models could be beneficial for better understanding of the stromal and cancer cell behaviors and cell-cell interactions [183], [184], [185]. Hanging drop technique has been utilized previously to create 3D tumors *in vitro*. Most pancreatic cancer cells do not form cohesive and compact spheres when the original hanging drop method is used. However, a modified hanging drop method following addition of methylcellulose polymer was capable of growing uniform and reproducible spheroids for all five of the tested human pancreatic cancer cell lines; Panc-1, BxPC-3, Capan-1, MiaPaCa-2, and AsPC-1 [186]. Tanaka et al. developed 3D bioprinted constructs of liver fibrosis model by using pancreatic stellate cells (PSCs), main resident cells of pancreatic cancer desmoplasia. In these 3D pancreatic tumor models, there was a significant improvement in collagen deposition and orientation of fibronectin fibril which was considerably remodeled by the influence of PSCs without any impact on fibroblasts [187]. Cancer-associated fibroblasts (CAFs) found frequently in tumor microenvironment have a definite function in tumor development and drug efficacy [188]. Hou et al. in their study, fabricated tumor models using two types of CAFs and two different pancreatic cancer cells derived from human pancreatic tumor tissue and were able to produce organoids in the absence of ECMs using the 3D bioprinting technology [189]. Another study used magnetic force dependent bioprinting technology for the development of high-throughput screening (HTS) pancreatic 3D cancer constructs using a non-adherent standard flat-bottom well cell culture plates [190]. Oregon Health & Science University filed a patent on the development of bioprinted tumor model composed of both stromal and tumor equivalents [23]. Hakobyan et al. developed 3D pancreatic cell spheroid platforms employing laser-assisted bioprinting and exemplified their morphological changes over time through image analysis and phenotypic characterization [191]. The initial stage of PDAC development can be replicated using bioprinted spheroids based on a combination of acinar and ductal cells. Overall, standardized and optimized printing parameters are important to adapt successful 3D printed tissues and organs, which can have a significant role in personalized drug development process. Continuing challenges of 3D bioprinting, comprising biocompatible material requirements, suitable cell sources, neovascularization, and autonomous maturation with continuous functionality of the constructs, still needs to be improved for its wider clinical application.

### 5.7 Bioprinted ovarian cancer models

**Fig. 5:**
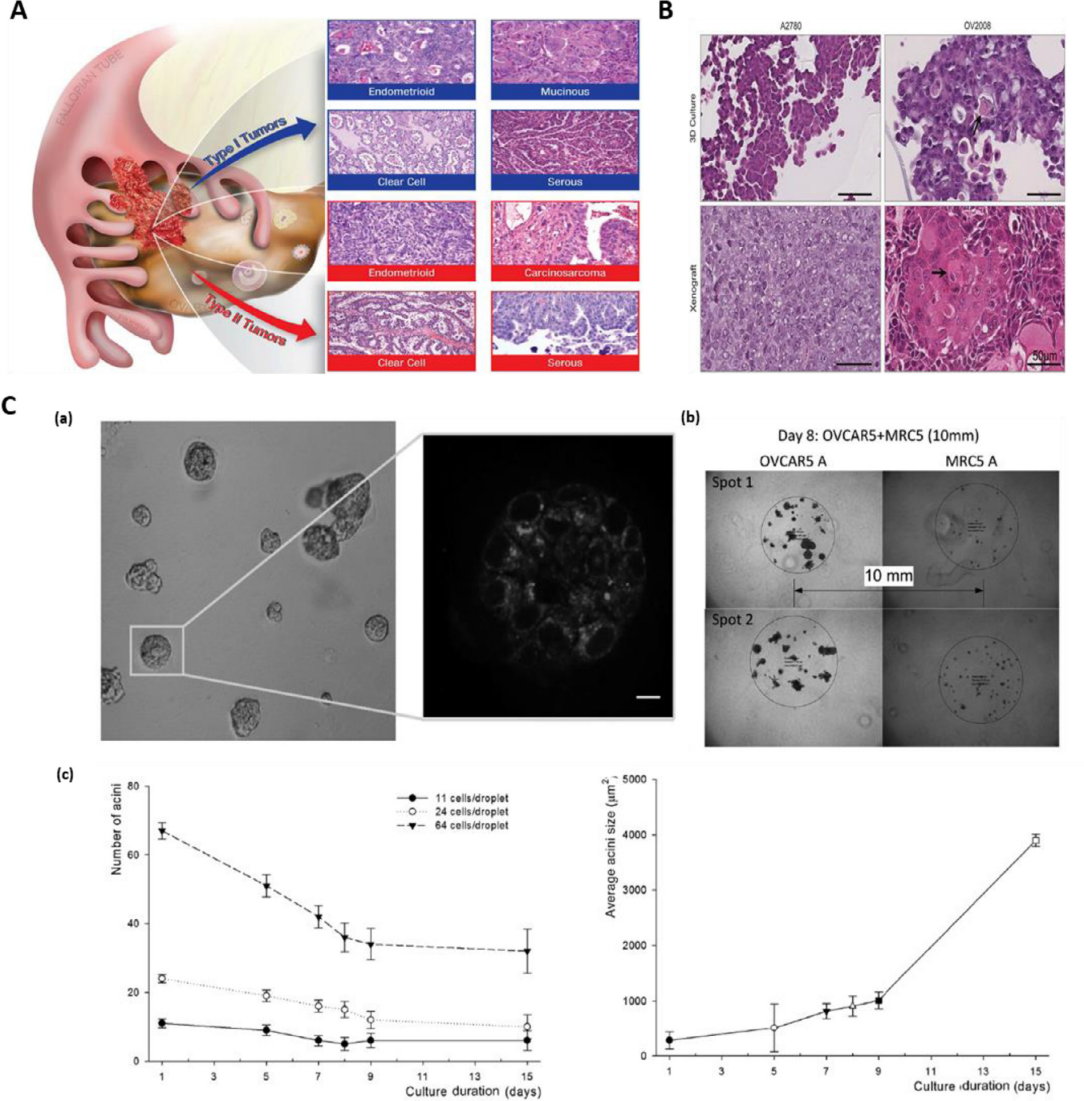
**Bioprinted ovarian cancer model: A.** The major subtype of ovarian neoplasm histologically divided in two subclassifications: Type I ovarian cancer contains low-grade, slow developing neoplastic cells that genuinely comes from boarder line tumors, which itself arises from ovarian surface epithelium, cysts, or endometriosis. On the other hand, Type II neoplastic cells are high-grade and fast developing. Typically, at the time of diagnosis this type is completely diffused beyond the ovary. **B.** Histology of A2780 and OV2008 cells when grown in 3D and as xenografts. Histological features of 3D and xenografted cells are highly similar. **C.** GFR Matrigel with formation of 3D acini ™. Printed OVCAR5 cells on Matrigel™ matrix and growth in culture medium. **(a)** Images of 7 days later after printing showing two photon autofluorescence with 3D structure of 3D acini formed by ovarian neoplastic cells. **(b)** Image showing the OVCAR5 and MRC-5 cells at 8^th^ day of coculture. **(c)** Acini growth kinetics after patterning: the number of acini changes as a function of initial number of cells per droplet. Average acini size increases with culture time. Figure A is reproduced from Ref. [196] with Creative Commons Attribution license (CC-BY). Fig. B is reproduced from Ref. [198] with the permission of Springer Nature. Fig. C is reproduced from Ref. [197] with the permission of Wiley.

Ovarian cancer is the seventh most lethal cancer in women [192]. High grade serous ovarian cancer (HGSOC) is the most widespread and aggressive form leading to the majority of advanced ovarian cancer cases [193]. Despite improvements in diagnosis and treatments, 5 years survival rate in ovarian cancer patients is still very low, ∼30% [194] Overall, there are two main types of ovarian cancers classified as Type I and Type II (Fig. 5-A,B). Among the subtypes of ovarian cancer cells, epithelial tumors are known to have low malignant potential; this subtype is more common in young women. In type-I epithelial ovarian cancer type, precursor lesions are clearly defined, whereas in Type-II lesions, malignant cells may arise from the tubal and/or ovarian surface epithelium [195]. The discovery of tumor diversity and subsequent advances in genomic technologies over the last few years have led to molecular classification of ovarian cancer that have informed the design of new experimental models including 3D bioengineered systems, for early and accurate detection and to develop better therapeutic interventions [105,196]. An example of this is the use of MRC-5 fibroblasts and human ovarian cancer (OVCAR-5) cell models that were bioprinted by Droplet-based bioprinting (DBB) on Matrigel to form high-throughput, reproducible multicellular constructs with controlled spatial environment (Fig. 5-C) This approach thus provided a platform to minimize the size of macro-scale 3D culture model and can serve as an innovative platform comprising tumor and stromal cells microenvironments for a high-throughput and robust personalized drug screening [197]. Lee et al. developed 3D ovarian *in vitro* tumor model composed of epithelial ovarian cancer (EOC) cells, reporting a substantial transition in histological features of 3D constructs compared to 2D tumor models, which are hallmark features of primary tumors.

### 5.8 Bioprinted lung cancer models

Based on histological classification, there are two main types of lung cancers- I) small cell lung cancer and II) Non-small cell lung cancer [199]. For advance management and estimating the prognosis, these classifications are very important. Adenocarcinoma comprise approximately 40% of lung cancers, which arises from peripheral lung tissue. Long-term smoking and other environmental contaminants are the main causes of lung cancer. Despite substantial advances in early diagnosis and improved interventions, the 5-year survival rate is only 15% [200]. Therefore, there is an unmet clinical need for the development of better diagnostic and treatment strategies. In order to achieve this, better understanding of the molecular mechanisms are required especially in terms of cancer cell signaling. 3D tumor models of adenocarcinoma containing decellularized extracellular matrices and hydrogels formed commonly from collagen and alginate, or other polymers have been used in lung cancer studies for these purposes [201]. These 2D and 3D tumor models produce distinct phenotypic and genotypic changes in cells. Mazzocchi et al. established 3D tumor models with lung cancer spheroids embedded within hydrogel scaffolds and encapsulating the specific cell types and pleural effusion aspirates from biopsy samples from a large number of lung cancer patients [202]. It was also demonstrated that placing the cells into organoids created anatomically relevant structures in addition to showing adenocarcinoma specific behaviors. An interesting study indicated that tuning of rheological properties of sodium alginate-gelatin hydrogel improves the printability and viability of non-small cell lung cancer patient derived xenograft cells and cancer associated fibroblasts co-cultures, allowing for 3D spheroid development within the printed construct [203]. The spheroids exhibited tumor-specific markers (e.g. vimentin and α-SMA), indicating cellular crosstalk and its potential application in high-throughput drug screenings. Wang et al. used 3D bioprinted scaffolds to study lung cancer metastasis formed from gelatin–sodium alginate laden with A549/95-D lung cancer cells [204]. Thus, bioprinted tumor models developed using organoids from patient-derived tumor cells of pleural effusion fluid exhibit in-vivo-like anatomy. Such new cancer models can be utilized for understanding the pathogenesis of the lung cancer, test various drug combinations and novel therapies as well as identify specific biomarkers of response. All of these will lead to precision medicine and better outcomes for the patients.

### 5.9 Bioprinted liver cancer models

**Fig. 6:**
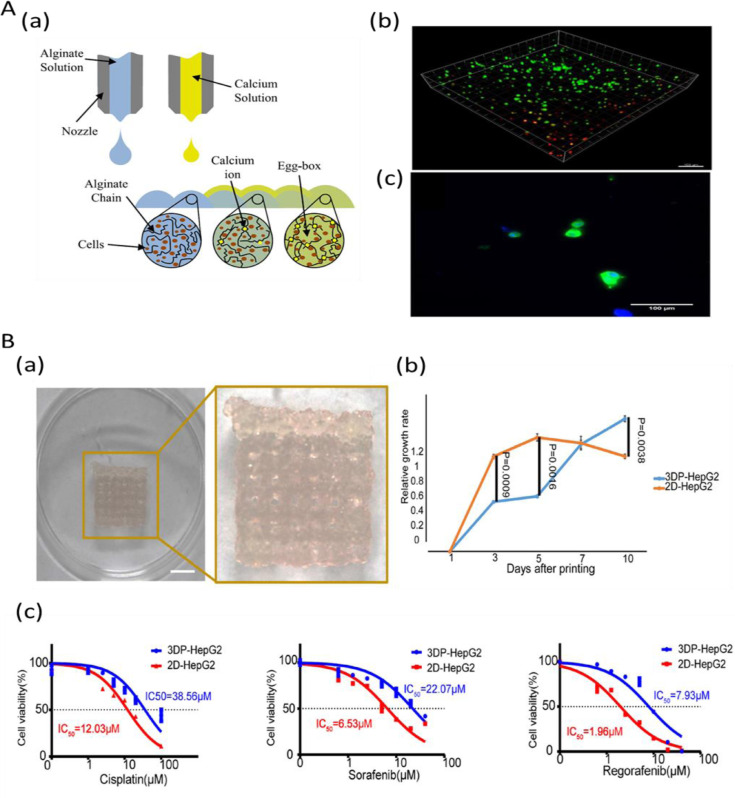
Bioprinted liver cancer models: A. (a) Schematic of the combinatorial printing process for Alginate hydrogel and hepacyte cells. (b) Fluorescence images of bioprinted hESC-derived hepatocytes: a 3D stack of hepatocyte-like cells printed in hydrogel, measured one hour after printing displaying live cells in green and dead cells in red (c) 2D cells collected from the alginate at day-23 indicating albumin expression in green and cell nuclei (DAPI) in blue. B. (a) The bioprinted model of hepatocellular carcinoma (3DP-HepG2) directly after printing (b) Proliferation rates of cells in 3DP-HepG2 and in its 2D counterpart (2D-HepG2) at different time points. (c) Dose-effect curves of cisplatin, sorafenib, and regorafenib in the 3DP-HepG2 and 2D-HepG2 models post-72 h drug treatment. Fig. A is reproduced from Ref. [209] under creative commons attribution license (CC-BY-3.0). Fig. B is reproduced from Ref. [212] under creative commons license (CC-BY-4.0).

Hepato-cellular carcinoma (HCC), the fifth most common malignant cancer represents about 90% of primary liver cancers and constitutes a major global health problem [205]. For all stages combined, the survival rate of liver cancer patients is ∼ 18% [200]. Liver cancer has a poor prognosis owing to delayed diagnosis and thus limited treatment options. Rodent models do not fully simulate the complex human cancer phenotypes due to variations in hepatocellular functions of different species, impeding the progress in drug development for this cancer [206,207]. Furthermore, there is also a lack of models that accurately represent patient-to-patient variation and heterogeneity of human liver cancer tissues. Various 3D bioprinting approaches have been used to develop biomimetic liver tissue constructs that can recapitulate native liver tissue microenvironment, vasculature and precise spatiotemporal signaling [208,209]. Initial studies focused on the printing of 3D constructs encapsulated with hepatocyte-like cells in hydrogel bioinks (e.g. calcium crosslinked alginate) to generate 3D liver tissue constructs (Fig. 6A). Since liver disease progression and chemotherapeutic drug response vary between individuals personalized *in vitro* human liver cancer model is a highly promising approach to better understand the disease mechanism, and serve as a drug screening platform, eventually improving the treatment of disease [210]. An interesting study that used rapid light-based 3D bioprinting process, a liver decellularized ECM-based construct for HCC progression in a cirrhotic mechanical environment showed a higher expression of invasion associated markers in the HepG2 cells of bioprinted model [211]. In a study by Sun et al., a 3D bioprinted model with HepG2 cells was developed and compared with 2D cultured tumor cells [212]. Results showed a remarkable improvement in the expression of tumor-related genes including ALB, AFP, CD133, IL-8, EpCAM, CD24, and TGF-β genes in 3D bioprinted model. Also, large variations in drug resistance genes in response to antitumor drugs and differences in gene expression related to hepatocyte function and tumor was observed between bioprinted models and 2D counterparts (Fig. 6B). A bioprinted tumor construct developed using patient-derived intrahepatic cholangiocarcinoma cells and gelatin-alginate-Matrigel^TM^ showed colony forming ability, high proliferation and survival [78]. Gene expression studies of the constructs showed the presence of higher level of cancer/stemness associated cellular markers, matrix metalloproteinase and EMT regulatory proteins indicating the formation of an invasive and metastatic type of cancer microenvironment in the construct. Likewise, a 3D bioprinted liver cancer model comprising of HepG2 cells and sodium alginate/gelatin/fibrinogen hydrogel was developed [213]. Further, upon treatment with different anti-cancer drugs, such as 5-Fluorouracil, mitomycin and both, 3D models exhibited substantially different HepG2 cell behaviors compared to 2D cell models indicating the closer physiological relevance of bioprinted liver cancer models for *in vitro* drug screening. A novel 3D biomimetic HCC model composes fibrotic stromal compartment and vasculature was developed, which can mimic bio-physical properties of a fibrotic, cirrhotic and HCC liver. This model showed chemotherapeutic drug resistance commonly seen in HCC patients. This 3D tumor construct could provide a reliable new platform to investigate multifocal HCC that contribute to early stages of cancer metastasis [214]. Also, a 3D printing model of intrahepatic vessel was developed with the application in a navigation surgery of hepatocellular carcinoma and provided an early proof-of-concept for further development of vessel like structures in bioprinted liver cancer constructs [215]. Both HCC and heterogeneous HCC/human umbilical vein endothelial cells combinations were bioprinted in multi-well plates to utilize the advantage of *in vivo* tumor heterogeneity and high throughput chemotherapeutic testing [112]. These studies indicate important potential applications of 3D bioprinting technology in liver-related biomedical fields for studying drug discovery, toxicology, and other pre-clinical applications.

## 6 Challenges and prospects

Limitations of current *in vitro* and *in vivo* cancer models to recapitulate the genetic makeup, molecular biology, and physiology of cancer cells in human tumour tissues impedes their applicability in the screening of chemotherapeutic drugs and precision medicine. Despite significant progress that has been made in the development of a number of new anti-cancer therapies using traditional cancer models, these systems have limited applicability in screening of personalized treatments as these are unable to reliably reproduce the heterogeneity of tumour tissue and the variation in cancer molecular and cellular characteristics from individual patients. In this context, 3D constructs containing patient-derived cells that are propagated *in vitro* within similar microenvironment as human *in vivo* tumours, are more reliable for generating personalized disease models. Although there has been a substantial progress and emerging potential of bioprinted cancer models for these applications, a number of challenges still remain, limiting translation to clinical and industrial applications. Although large scale manufacturing of bioprinted constructs is still in its infancy, manufacturers including Cellink and their partners, Prellis Biologics, are in the process of developing bioprinting equipment that can potentially address the requirements of industry and the clinic. Holograph X bioprinter is intended to facilitate the in-lab manufacturing of vascularized tissue constructs for organ transplant, via holographic projection printing. Bioprinting of patient-derived tumour constructs for screening of anticancer agents for the purpose of precision medicine is still in the development and it will take a number of years before it can be used in the clinical setting. From the very beginning, these technologies should comply with the good manufacturing practice (GMP) standards and the regulatory requirements, in order to standardize and optimize the bioprinting process of patient-specific tumours. Future studies should focus on the development, fabrication, and application of bioprinted tumour constructs in compliance with GMP standards. These bioprinted platforms should also be adapted accordingly for high throughput screening of chemotherapy and other anti-cancer agents by taking into the account the ease of use and the cost. Hence, future research should also focus on the rapid and automated determination of cellular parameters including cell viability and cell cycle within the 3D construct in real-time. Integration of organotypic constructs within simulated physiology of microfluidic platforms are challenging with the potential to incorporate all the required characteristics of human *in vivo* tumours.

Published evidence in the field of bioprinted cancer models indicates that bioprinted cancer models are highly promising platforms for evaluation of personalized anti-cancer treatments. Nevertheless, there are still substantial gaps in the knowledge in terms of various aspects of tumour bioprinting including rapid fabrication of 3D cancer models, their maintenance *in vitro* and cost-effectiveness for high-throughput screening and application in the low resourced clinical setting. The design and development of a fully integrated bioprinting mechanism is key for the commercial translation of bioprinting technology for precision medicine purposes in small to medium size laboratories. Future studies should focus on the approaches that help to miniaturize the bioprinted constructs suitable for automated workflow systems that are less labour -intensive and cost-effective.

The accurate control over physicomechanical and biological characteristics of a bioprinted construct is possible using advanced 3D printing technologies. Suitable material for bioprinting is required to precisely be placed with the preferred spatial and temporal control. Bioprinting technologies like inkjet have drawbacks in terms of material viscosity whereas others such as microextrusion may need materials having specific crosslinking mechanisms or shear-thinning properties. Nozzle gauge is one of the processing parameters determining the shear stress to which cells are exposed to along with the time necessary for the material to be placed to produce a 3D structure. For instance, inkjet printing involves the use of materials with a rapid crosslinking time to enable the generation of a complex 3D structure whereas microextrusion can make use of highly viscous materials to keep a 3D shape after deposition with final crosslinking that occurs following fabrication. A recent report indicated that thermal inkjet bioprinting has impact on gene expression including those involved in key drug resistance, cell motility, proliferation, cell survival, and differentiation. These are important factors to consider and monitor as part of the process of bioengineering tumour constructs by bioprinting.

The cell viability, a critical factor for cancer modelling, can be affected by the shear forces generated during extrusion bioprinting. Recent approaches focusing on LAB methods may be a solution for maintaining cell viability in the future, bearing in mind that photoinitiator and UV light used in LAB need further optimising to minimise their effect on cell viability. Less toxic photoinitiator or visible light photocrosslinking approaches may solve these issues. Several other crosslinking approaches are also used for bioprinting to provide the sufficient mechanical strength and relatively long degradation rate to the bioprinted construct. A wide range of natural polymers including gelatin, alginate, chitosan, and collagen, have been used as a bioink for printing tumour constructs because of simplicity of their crosslinking mechanisms. However, ionic, or chemical crosslinking agents used for hydrogel systems can also affect cell viability and phenotype of the tumour construct. Recent advancements with introduction of photocrosslinkable methacryloyl groups into these biopolymers is showing some progress towards better preservation of cell viability and phenotype. However, fine tuning the methodologies for bioprinting cancer and stromal bioinks capable of recapitulating the properties of physiological tumour tissues and their microenvironment in adequate spatial and temporal manner needs to be further explored. These models can also recapitulate the heterogeneity of the tumors using different materials and cells simultaneously within the same bioprinted construct. As a drug screening platform, bioprinting should be applicable to several cancer types including breast cancer, GBM, cervical cancer, lung cancer and ovarian cancer.

One of the key challenges in cancer bioprinting is the difficulty in developing well-established vascular network within tumors, which is necessary given that tumor tissues contains large number of highly branched capillary networks that facilitate tumour growth. Thus, connecting the bioengineered tumour tissue with surrounding stromal tissue is necessary for mimicking these cellular communications that are present *in vivo*. Generating bioprinted constructs with well-developed vasculature is particularly important in GBM, tumor characterized by pronounced angiogenesis. Unsurprisingly, a study investigating angiogenesis within MCTS of undifferentiated melanoma cell line, NA8-MCTS, revealed that the invasion and network formation of HMEC-1 cell line within NA8-MCTS increased following co-culturing with HMEC-1 [149]. As mentioned above, it has been reported that a subtype of glial stem cells within the tumor are able to trans-differentiate into endothelial cells promoting angiogenesis [150], therefore utilizing these patient-derived cancer cells may establish a vasculature in bioprinted cancer construct in the presence of adequate tumor microenvironment. Although various vascularization approaches are used, this could be achieved by introducing channels inside the biofabricated tissues and allowing media to perfuse the construct and facilitate endothelial cells and smooth muscle cells to adhere and proliferate on the walls of channels. Nevertheless, vascularization approaches still need to be investigated further and optimized for specific tumour types. On the other hand, within human tumor *in vivo* environment, cellular secretion of enzymes, hormones, cytokines, or other agents can influence the microenvironment of the growing tumour. Hence, all these cellular and molecular factors need to be taken into the account when designing and bioprinting tumor constructs.

Although the bioprinted cancer models share a common 3D conformation, each display its own intrinsic properties. For example, a variety of *in vitro* and *in vivo* findings have demonstrated the significance of the interaction between the tumour cells and host stromal cells including endothelial cells, fibroblasts, adipocytes, mature immune cells, and circulating immune cell progenitors. Incorporation of various types of healthy cells and specific cancer cells is a major challenge since it is far from simply combining similar types of cells. However, recapitulating cellular communications is vital for paracrine and autocrine molecular signalling that affects key hallmarks of cancer. These include interactions with the surrounding matrix, host cells, and receptors or ligands of both cancer and stromal cells. For instance, CAFs that affect tumor microenvironment are the most common component of cancer stroma mainly in breast and pancreatic cancer, MRC-5 fibroblasts and OVCAR-5 in human ovarian cancer cell models, a set of cancer and stromal cells including U87MG glioblastoma cells, MM6 monocyte/macrophages, glioblastoma stem cells, glioma associated stromal cells (GASCs) in GBM and iPSCs in colorectal cancer models have been used for personalized drug screening. Understanding biology of specific types of tumors well will aid the development of suitable bioprinted platforms with high predictive power, capable of drugs screening and precision medicine.

The effectiveness and purpose of tissue constructs can be designed, and hence improved, before it is printed using computer models. Therefore, design stage is key for generating optimal and specific 3D cancer tissues representative of physiological tumours. Rapid developments in bioprinting instrumentation along with progress in bioink preparation and bioprinting process can significantly improve the ability of the construct to closely mimic cancer tissue and its reliability to test chemotherapeutic drug efficacy. Moreover, regulatory agencies should have standardized guidelines for the requirement of processes and procedures to accelerate the clinical translation of these bioprinted tissue models and enable personalized treatments that will improve outcomes for the patients.

## 7 Conclusions

Over the last two decades, the tools used to create 3D cell cultures, organoids, and other 3D *in vitro* models, including cell supportive biological material and 3D bioprinting, have rapidly progressed. Conventional cancer models have a number of limitations due to the lack of capacity to mimic the complexity of tumours, thus restricting their utilisation for chemotherapeutic drug screening. Attempts to diligently mimic the tumour microenvironment appear to be feasible with the introduction of latest technologies such as bioprinting and microfluidic chips. Past research shows that the 3D bioprinting has a huge potential to construct tissue models that can recapitulate tumour microenvironment at molecular, cellular, and physiological level. Bioprinted breast, brain, skin, colorectal, pancreatic, and other cancer models were highly successful in mimicking tumour anatomy, physiology, and molecular biology. Patient-derived 3D bioprinted cancer models could be successfully used for *in vitro* drug screening of anti-cancer drugs in order to develop personalized treatments. Ongoing and future studies are focusing on the development of integrated microfluidic/bioprinted constructs in a lab-on-a-chip format to minimize the cost and facilitating high throughput testing of a large number of available chemotherapeutic agents enabling selection of the most suitable drug combination for a particular patient and hence personalized medicine approach.

## Author Contributions

Conceptualization: R.A (Robin Augustine). Investigation: R.A (Robin Augustine)., N.K.S., Resources: R.A. (Robin Augustine). Writing—original draft preparation: R.A (Robin Augustine)., N.K.S. Writing—review and editing: R.A (Robin Augustine)., N.K.S., R.A (Rashid Ahmed)., A.N., L.M., A.A.Z. Visualization: N.K.S., R.A (Robin Augustine). Supervision: R.A., A.H. Project coordination: R.A. Funding acquisition: A.H. All authors have read and agreed to the published version of the manuscript.

## Declaration of Competing Interest

Authors declare that there is no financial/personal interest or belief that could affect the results, discussions or conclusions which are reported in this work.
